# Research Progress on Light Therapy to Improve Depressive Symptoms of Bipolar Depression

**DOI:** 10.62641/aep.v54i4.2255

**Published:** 2026-08-15

**Authors:** Yutong Cui

**Affiliations:** ^1^College of International Business and Economics, Wuhan Textile University, 430202 Wuhan, Hubei, China

**Keywords:** light therapy, bipolar disorder, bipolar depression, depressive symptoms, circadian rhythm

## Abstract

Bipolar depression is the predominant and most disabling phase of bipolar disorder, featured by recurrent depressive episodes that impair psychosocial function and quality of life. Conventional pharmacotherapy has limited efficacy and carries a risk of mood destabilization (e.g., affective switch). Light therapy, a nonpharmacological chronotherapeutic intervention, has shown potential to improve bipolar depression symptoms by regulating circadian rhythms and mood regulating neural circuits. This review synthesized domestic and international research advances regarding light therapy for bipolar depression, focusing on the mechanisms of action, clinical efficacy, application protocols, safety, and limitations, aiming to provide evidence based clinical guidance for optimization of therapeutic strategies.

## Introduction

Bipolar disorder is a chronic psychiatric condition characterized by alternating episodes of hypomania or mania, depression, and mixed states, along with persistent subsyndromal symptoms that contribute to functional impairment [1]. The disorder frequently co-occurs with multiple psychiatric and medical comorbidities, complicating its clinical management. It exhibits high heritability, typically emerges in adolescence or early adulthood, and is strongly associated with co-occurring anxiety and substance use disorders. These factors collectively lead to substantial psychosocial disability and markedly reduce patients’ overall quality of life [2]. Among the illness phases, depression is the most common and disabling, accounting for approximately 75% of symptomatic periods in many individuals [3].

This depressive phase—bipolar depression—is defined by persistent low mood, anhedonia, psychomotor disturbances and cognitive impairment. These symptoms not only reflect the underlying mood pathology but also serve as prodromal indicators and significant risk factors for episode recurrence [4]. Although conventional pharmacotherapies remain the mainstay of treatment, their efficacy in addressing core depressive symptoms is often suboptimal, and they carry a notable risk of mood destabilization (e.g., affective switch), and may even increase the risk of cardiovascular adverse events [5, 6]. This therapeutic gap underscores the urgent need for novel, well-tolerated interventions. In this context, light therapy has emerged as a promising nonpharmacological chronotherapeutic strategy that targets circadian rhythm regulation to improve depressive symptomatology in bipolar disorder.

Light is the primary environmental cue (zeitgeber) for synchronizing the human circadian system, which governs hormone secretion and mood. However, modern lifestyles involve prolonged indoor periods, where architectural features filter and attenuate natural daylight [7], combined with pervasive evening exposure to artificial and screen-based light [8]. This pattern of suboptimal daytime light and mistimed evening light causes circadian misalignment, a condition implicated in various mood disorders. Light therapy is an affordable, nonpharmacological intervention that uses specific light wavelengths to treat various medical and psychological conditions [9] and has been clinically validated as effective for bipolar depression [10]. The proposed mechanism involves daily morning exposure to high-intensity light that stimulates retinal cells, activates the circadian system, phase advances circadian rhythms, and increases brain arousal, thereby alleviating depressive symptoms in bipolar depression [11].

Given the clinical effect of depressive symptoms in bipolar depression, along with the limitations and mood-destabilizing risks of conventional pharmacotherapy, light therapy represents a promising nonpharmacological chronotherapeutic intervention. Therefore, this review systematically summarized current evidence on the efficacy, mechanisms and clinical application of light therapy for improving depressive symptoms in patients with bipolar depression.

### Search Strategy and Study Selection

To identify relevant studies, we systematically searched PubMed, Web of Science, Cochrane Library and Embase from inception to 2026 using keywords including “bipolar disorder”, “bipolar depression”, “light therapy”, “bright light therapy”, “phototherapy”, “circadian rhythm”, and “depressive symptoms”. The inclusion criteria were as follows: (1) randomized controlled trials, quasi-experimental studies, or open-label trials with a comparison group that investigated light therapy (including bright light therapy, morning light treatment, dawn simulation, or blue-enriched light) in individuals with bipolar depression and evaluated the effects on depressive symptoms; (2) systematic reviews or narrative reviews that synthesized evidence or provided clinical/mechanistic background on light therapy for bipolar depression; and (3) peer reviewed articles published in English. The exclusion criteria were as follows: (1) original studies focusing exclusively on unipolar depression, seasonal affective disorder, or other depressive subtypes without reporting separate data for bipolar depression; or (2) studies whose primary outcome was sleep related measures rather than depressive symptom severity.

## Mechanism of Action of Light Therapy

The suprachiasmatic nucleus (SCN) is the centre regulator of all circadian rhythms, including sleep–wake cycles, appetite, autonomic nervous system and neuroendocrine rhythms [12]; it is a small nucleus containing approximately 20,000 neurons [13]. The circadian timing system relies on the photopigment melanopsin (OPN4), not the visual photoreceptor rhodopsin, to transmit light signals via intrinsically photosensitive retinal ganglion cells (ipRGCs) through the retinohypothalamic tract to the SCN [14]. Within the SCN, arginine vasopressin (AVP)-producing neurons reside in the shell (dorsomedial region), and vasoactive intestinal polypeptide (VIP)-producing neurons reside in the core (ventrolateral region). AVP neurons release gamma-aminobutyric acid (GABA), which suppresses intermediate GABAergic neurons located between the shell and core; this disinhibition indirectly activates VIP neurons. This GABAergic network from AVP to VIP neurons precisely sets the timing of the behavioural activity/rest period. Conversely, VIP neurons do not provide essential GABAergic feedback to AVP neurons for network synchrony [15]. This input enables the SCN to align its internal timing with the external light-dark cycle, thereby regulating daily physiological rhythms. Circadian rhythms are endogenous cycle of appropriately 24 hours that regulate numerous physiological and behavioural functions, including sleep–wake cycles, hormone secretion, metabolism and feeding behaviour [16]. Notably, disruption of this system is closely associated with the onset and progression of bipolar depression, and light therapy can improve depressive symptoms by restoring this pathway.

The SCN regulates the timing of most circadian rhythms, including the daily release of various metabolism-related hormones. The best-known SCN-mediated endocrine response is the production and secretion of melatonin by the pineal gland [17]. Melatonin is a nocturnal hormone that acts as a biochemical clock, regulating numerous physiological and behavioural processes such as sleep–wake rhythms, seasonal adaptation, hormone secretion, thermoregulation, reproduction and digestion, thereby reflecting circadian rhythm [18]. Research indicates that bipolar depression is associated with significantly reduced melatonin secretion and altered circadian rhythms [19, 20], in which dysfunctions in the melatonin biosynthesis pathway [20] and melatonin receptor type 1 (MT1) receptor signalling [21] play a critical role in the disorder’s pathophysiology and symptom manifestation. Therefore, patients with bipolar disorder exhibit melatonin secretion abnormalities, and light therapy can indirectly modulate melatonin secretion by regulating the SCN, thereby helping to normalize circadian rhythms.

Circadian rhythm disruption, involving the ipRGC SCN photic input pathway, is associated with mood instability and depressive symptomatology in bipolar disorder; however, the current evidence does not establish this disruption as a core pathophysiological mechanism, and further research is needed to clarify its causal role and specificity [22]. Nonetheless, persistent circadian misalignment has been associated with increased recurrence rates of mood episodes [22], and chronotherapeutic approaches that stabilize and normalize circadian rhythmicity are effective in preventing mood episode relapses [23].

Beyond SCN mediated circadian entrainment, bright light exposure directly modulates serotonergic systems in mood-regulating brain regions (e.g., prefrontal cortex) and dopaminergic systems via pathways independent of circadian phase shifting [24]. Additionally, photobiomodulation—using lower intensity red or near infrared light—may influence mitochondrial function, reduce oxidative stress, and enhance cerebral blood flow, offering parallel non circadian mechanisms that alleviate depressive symptoms and may lower relapse risk [25].

In summary, light therapy influences circadian rhythms in bipolar depression through multiple pathways: it regulates melatonin secretion via SCN entrainment; it directly modulates serotonergic and dopaminergic systems in mood-regulating brain regions; and it may reduce oxidative stress and enhance cerebral blood flow through photobiomodulation. These integrated mechanisms—circadian, neurotransmitter, and metabolic—collectively alleviate depressive symptoms.

## Clinical Application of Light Therapy in Improving Symptoms of Bipolar Depression

As a key regulator of circadian rhythms, light has attracted considerable research attention in recent years for its therapeutic potential in treating depression and related conditions [26]. Its ability to directly influence the brain’s internal clock makes it a promising nonpharmacological intervention. These benefits are thought to arise from stabilizing circadian timing and increasing daytime alertness. Furthermore, a 12-month prospective cohort study of 202 euthymic patients with bipolar disorder found that greater objective daytime light exposure, particularly in the morning, was significantly associated with a reduced risk of depressive episodes relapse (hazard ratios ranging from 0.61 to 0.66). Although causality cannot be definitively established due to the observational design, these findings suggest a potential prophylactic role for bright light exposure in the long-term management of bipolar depression [27]. However, as an observational study with a moderate sample size, residual confounding cannot be excluded, and the absence of a control group limits causal inference. Furthermore, recent real-world evidence, including patients with bipolar I and bipolar II disorder, demonstrated that add-on bright light therapy administered for one to two weeks was associated with significant reductions in self-reported depressive symptoms, with effect sizes comparable between bipolar and unipolar depression [28]. This naturalistic study lacked a placebo control and blinding, used a flexible treatment duration prone to selection bias, had a small bipolar sample (n = 41), and included no long-term follow-up.

A review of nonpharmacological interventions indicates that intensive light therapy is a promising candidate for treating bipolar depression, potentially aiding in mood stabilization and symptom reduction [29]. However, given the limited number of high-quality randomized controlled trials specifically targeting bipolar depression, the evidence base remains preliminary. Moreover, a systematic review of randomized controlled trials confirmed that adjunctive bright light therapy significantly reduces depressive symptoms in bipolar depression without reports of serious adverse events or mood switches [30]. The meta-analysis revealed a medium-to-large post-treatment effect size (Hedge’s g = −0.74; 95% CI: −1.05 to −0.42; *p* < 0.001) across six included studies [30]. However, the existing evidence base is limited by the small number of studies per intervention, inconsistent protocols, and a lack of long-term follow-up data, highlighting the need for further high-quality trials. Consequently, the overall certainty of evidence remains moderate at best, and these findings should be interpreted cautiously pending larger, more rigorously designed trials.

### Treatment Time

Study has shown that morning light therapy trials relieve depressive mood to some extent [31]. Moreover, a 2025 dose-escalation randomized controlled trial in patients with bipolar depression who were stably medicated with mood stabilizers found that 45 minutes of bright light therapy was well tolerated and effective. There was no significant difference in hypomanic switch risk between morning and midday administration. However, the morning bright light therapy group showed significantly greater improvement on the Clinical Global Impressions–Improvement scale, suggesting a potential efficacy advantage for morning timing [32]. A comprehensive review also concluded that morning or midday administration produces the most consistent antidepressant effects in bipolar disorder, with morning exposure being commonly used in clinical trials [33]. Thus, morning light exposure (45–60 minutes) appears to be the preferred timing for optimizing antidepressant effects in bipolar depression, with an acceptable safety profile when combined with mood stabilizers.

### Equipment and Methods of Light Therapy

Light therapy has specific and well-defined equipment requirements to ensure efficacy and safety. Treatment typically uses professional light box devices that deliver a calibrated light intensity of 10,000 lux [34]. This light intensity helps maintain a consistent and adequate irradiation dose despite slight patient movements. According to expert consensus guidelines for light therapy devices, white light is the recommended standard spectrum, as full-spectrum or blue-enriched lights are not superior and cause increased visual glare [35]. Devices must include an ultraviolet filter to eliminate potentially harmful ultraviolet rays, thereby protecting the eyes and skin. To enhance comfort and reduce glare, the light box should be positioned slightly above the user’s eye level, allowing the light to project downward. However, the authors note that many commercial devices have not undergone clinical trials, and current evidence primarily supports light boxes that adhere to these technical specifications [35].

For bipolar depression, the standard light therapy regimen typically requires a longer daily session of 45–60 minutes to achieve therapeutic effects, as this condition involves more persistent circadian dysregulation than seasonal affective disorder. Regular morning outdoor activities offer a natural and effective alternative, as natural sunlight provides intensities far exceeding those of artificial light boxes, thereby serving as a convenient and cost-effective adjunctive treatment [35]. Furthermore, when combined with mood stabilizers, morning bright light therapy has been associated with significantly shorter hospital stays and a rapid antidepressant response, reflecting the synergistic effect of chronotherapeutic interventions in resetting the persistently dysregulated circadian system characteristic of this condition [36]. Thus, light therapy must be highly individualized: the optimal start time should be tailored to the patient’s circadian chronotype (e.g., “morning type” vs. “evening type”), which can be accurately assessed using tools such as the Morningness–Eveningness Questionnaire (MEQ) to match light exposure to the individual’s intrinsic rhythm. In summary, using a 10,000 lux white light box with a screen area ≥1000 cm^2^ for 45–60 minutes in the morning, combined with chronotype-guided timing, provides a standardized yet personalized protocol to effectively alleviate depressive symptoms in bipolar depression.

### Combined Treatment Regimen

Research has confirmed that chronotherapeutic packages integrating light with sleep deprivation are also effective. In a case series of 11 inpatients with bipolar depression, double chronotherapy (three cycles of total sleep deprivation over one week followed by daily light therapy) significantly reduced depressive symptoms by the end of the one-week intervention period, supporting its antidepressant efficacy in bipolar depression [37]. Another study found that combining chronotherapeutics (repeated total sleep deprivation and light therapy) with lithium was effective in treatment-resistant bipolar depression, producing a rapid antidepressant response in more than half of the patients [38]. Moreover, a case report demonstrated that triple chronotherapy was effective for a patient with bipolar depression, with no manic switch reported during the protocol and several years of follow up [39]. Collectively, these findings indicate that combining light therapy with medication (mood stabilizers) or other chronotherapeutic modalities enhances antidepressant response and may accelerate symptomatic improvement in bipolar depression while maintaining a favourable safety profile.

## Side Effects of Light Therapy

Light therapy is generally safe and well-tolerated. Common side effects (e.g., headache, eye strain, nausea, irritability) are mild and transient, typically subsiding within days. Evening administration may cause initial insomnia or hyperarousal [40]. To improve tolerability, start with shorter sessions (e.g., 15–30 minutes) and gradually increase. Contraindications include acute mania/hypomania, mixed episodes, rapid cycling, retinal diseases and use of photosensitizing medications [41]. In bipolar depression, light therapy is efficacious with a low risk of hypomanic switch—lower than that of conventional antidepressants—and should always be combined with a mood stabilizer, especially in bipolar I disorder [41]. Monitor for early warning signs (e.g., irritability, decreased need for sleep, pressured speech). Under appropriate safety protocols, light therapy is largely free of significant adverse effects.

## Research Limitations and Future Prospects

Several methodological limitations currently constrain the robustness and generalizability of findings in light therapy trials. Firstly, many studies have notable deficiencies in sample size and study design. Most randomized controlled trials in this field have relatively small sample sizes, typically fewer than 100 participants, which restricts statistical power and limits extrapolation of results to broader clinical populations. Secondly, some existing evidence comes from naturalistic or randomized controlled trials that often lack long-term follow-up data, hindering assessment of sustained therapeutic effects and potential relapse rates. Thirdly, many light therapy studies for bipolar depression rely heavily on subjective clinician- or self-rated depression scales, while insufficient use of objective neurobiological or functional outcome measures. This reliance on subjective reporting may introduce bias and reduce the precision of efficacy assessments. Fourthly, considerable heterogeneity exists across studies in light protocols and outcome measures, and inadequate control for concomitant medications further limits the overall certainty of evidence.

Future research should pursue the following directions: Firstly, high-quality clinical research: design large-sample, multicentre, long-term randomized controlled trials specifically for bipolar depression, stratifying patients by bipolar subtype (type I vs. type II) and polarity predominance to determine whether treatment response differs across subgroups. Incorporate long-term follow-up assessments to evaluate not only sustained antidepressant effects but also the risk of mood switch (mania/hypomania) and episode relapse rates, overcoming the lack of longitudinal data. Use objective neurobiological or functional outcome measures, such as neuroimaging biomarkers, alongside standardized depression scales to reduce reporting bias. Simultaneously, conducted head-to-head studies comparing the efficacy of different light parameters, equipment, and combination regimens, with careful documentation of concomitant mood stabilizer use, as lithium and other medications may influence light sensitivity and circadian responses. Furthermore, real-world study designs to include patients with comorbidities common in bipolar depression (e.g., anxiety disorders, metabolic syndrome) to evaluate the effectiveness and safety of light therapy in routine clinical practice.

Secondly, deepen research on the mechanism of action: combine multiomics technologies such as gene sequencing, brain functional imaging, and metabolomics to explore the association between core clock genes, neurotransmitter receptor genes and the therapeutic effects of light therapy, and construct a predictive model for efficacy. Simultaneously, through animal experiments and human studies, elucidate the details of how light therapy regulates neural circuits and molecular pathways, providing a theoretical basis for optimizing treatment regimens. Furthermore, further explore the regulatory effects of light therapy on auxiliary mechanisms such as inflammatory responses and oxidative stress, enriching its mechanism of action network.

Thirdly, promote equipment innovation and scenario expansion: develop smarter, more portable light therapy devices that integrate biofeedback and remote monitoring functions to enable dynamic adjustment of treatment parameters and intelligent management of clinical follow-up. Expand community- and home-based treatment scenarios, combining digital health technologies (such as app reminders and online guidance) to improve patient treatment adherence. Simultaneously, explore combining light therapy with novel treatment such as virtual reality and transcranial magnetic stimulation to further enhance efficacy. Furthermore, develop specialized devices for specific populations, such as low-intensity light therapy devices suitable for adolescents and intelligently controlled devices suitable for elderly patients.

## Conclusions

Light therapy, as a non-invasive physical therapy, has demonstrated promising efficacy in alleviating depressive symptoms during bipolar depression episodes, with a favourable safety profile and low risk of mood switch when combined with mood stabilizers. Based on the reviewed evidence, the following clinical recommendations can be made for bipolar depression: (1) morning bright light therapy at 10,000 lux for 45–60 minutes is recommended as an adjunct to mood stabilizers; (2) for patients at high risk of mood switch, midday exposure may be preferred over early-morning exposure; and (3) treatment should be individualized based on chronotype assessed by the MEQ, with close monitoring for early signs of mood elevation. Future high-quality, large-scale and well-controlled randomized trials with objective outcome measures are urgently needed to establish standardized treatment protocols and optimize the clinical application of light therapy for bipolar depression.

## Availability of Data and Materials

The datasets used and/or analysed during the current study were available from the corresponding author on reasonable request.
